# Effect of *Streblus asper* Leaf Extract on Scopolamine-Induced Memory Deficits in Zebrafish: The Model of Alzheimer's Disease

**DOI:** 10.1155/2021/6666726

**Published:** 2021-04-24

**Authors:** Kanathip Singsai, Natthanicha Ladpala, Natthan Dangja, Thanyaret Boonchuen, Niracha Jaikhamfu, Pirinyapat Fakthong

**Affiliations:** ^1^Department of Pharmaceutical Care, School of Pharmaceutical Sciences, University of Phayao, Phayao 56000, Thailand; ^2^Unit of Excellence of Pharmacological Research and Vaccine Development in Animal Models, University of Phayao, Phayao 56000, Thailand

## Abstract

*Streblus asper* (SA) is well known as a folk medicinal plant in Asian countries. The effect of SA extract on preventing memory impairment in zebrafish induced by scopolamine was investigated. Male zebrafish, *Danio rerio*, were divided into 6 groups including the control, scopolamine 200 *μ*M (SCO), scopolamine plus rivastigmine 1.5 mg/kg (RV + SCO), and scopolamine plus SA extract at doses of 200, 400, and 800 mg/kg (SA200 + SCO, S400 + SCO, and SA800 + SCO), respectively. Spatial memory was evaluated by the colour-biased appetite conditioning T-maze test, while fear memory was measured by the inhibitory avoidance test. In the spatial memory test, results showed that the RV + SCO group had the best time spent ratio in the T-maze, followed by SA800 + SCO, SA400 + SCO, SA200 + SCO, control, and SCO group, respectively, but with no statistical significance. For the fear memory test, zebrafish that received SA at doses of 200, 400, and 800 mg/kg had significantly increased latency time as 21.75 ± 4.59, 23.75 ± 13.01, and 18.20 ± 18.84 min, respectively, when compared to the SCO group (9.80 ± 10.45 min). These results suggested that SA extract attenuated memory impairment in an inhibitory avoidance test related to fear memory. Our findings can be useful for further research to develop SA extract as a health product to ameliorate the symptoms of Alzheimer's disease.

## 1. Introduction

Alzheimer's disease (AD) is a general term for loss of memory, thinking, and behaviour that interferes with daily life and activities. Most patients with AD are more than 65 years old. Worldwide, around 50 million people suffer from dementia, and nearly 10 million new cases are reported every year. More than 5 million Americans have Alzheimer's disease, with numbers of those over 65 living with the disease doubling every 5 years.

AD is a progressive neurodegenerative disorder. Histopathological characteristics are extracellular aggregation of amyloid *β* plaques (A*β* plaques) and intracellular aggregation of neurofibrillary tangles (NFTs) that consist of hyperphosphorylated microtubule-associated *τ* protein [1–3]. The formation of amyloid plaques stimulates microglia, known as neurotoxic microglia, to signalling via toll-like receptors (TLRs) and receptors for advanced glycation end products (RAGE). This induces transcription factors such as NF-*κ*B and AP-1 that generate ROS and induce the expression of inflammatory mediators such as cytokines (TNF-*α* and IL-1*β*), directly affecting cholinergic neurons as the primary neurons involved in learning and memory. The abnormal APP protein-controlled gene can induce excessive A*β* production. Zebrafish express two gene products, termed APPa and APPb, which are homologues of the human APP gene [4, 5]. The aggregation of hyperphosphorylated Tau can cause destabilized microtubules, resulting in neuron dysfunction. Two paralogues of MAPT in zebrafish are called mapta and maptb, and both mapta and maptb mRNAs were expressed in the developing CNS [6]. A*β* plaques and NFTs are promoted by acetylcholinesterase (AChE), an enzyme that hydrolyses acetylcholine as the neurotransmitter of cholinergic neurons. Acetylcholine (ACh) is involved in memory [7]. Currently available treatments are acetylcholinesterase inhibitors such as rivastigmine, galantamine, and donepezil for mild to moderate severity of Alzheimer' disease, and N-methyl D-aspartate receptor antagonist (NMDA) such as memantine for moderate to severe Alzheimer's disease. Treatment can temporarily slow the gradual onset of dementia symptoms and improve quality of life for patients with Alzheimer's disease [1].

Currently, most medicines that slow progression of disease are acquired from natural products. Plant extracts and phytochemicals are well known for their antioxidant, antimicrobial, and anti-inflammation properties, which can be of great importance in therapeutic treatments. *Streblus asper* (SA) is a natural product with many chemical properties. SA belongs to the family Moraceae and is a small tree which is indigenous to tropical countries such as India, Malaysia, and Thailand. Many studies reported different biological activities of SA in various in vitro and in vivo test models. Different parts of SA have been found to exhibit cardiotonic, antifilarial, anticancer, antimicrobial, antiallergic, antioxidant, antihyperglycemic, antimalarial, and anti-Parkinson activities [8, 9]. Some studies reported that SA has acetylcholinesterase (AChE) inhibition property which is beneficial for potential treatment of Alzheimer's disease (AD) [10].

This study examined the pharmacological properties of SA leaf extract to prevent scopolamine-induced learning deficits in zebrafish that share basic nervous system organization as models of human neurological disease [11]. The effects of SA extract on scopolamine-induced memory deficits in zebrafish were investigated to assess SA extract as a possible dietary supplement or medicine for the amelioration of the onset of Alzheimer's disease.

## 2. Materials and Methods

### 2.1. Preparation of Extracts

Leaves of *Streblus asper* were collected from the botanical garden of the School of Pharmaceutical Sciences, University of Phayao (Phayao, Thailand), washed, dried, and powdered coarsely using a blender. Maceration was employed for extraction using deionized water as a solvent at 60°C for 6 hours (100 grams of plant leaf per 1 liter of water). The filtrate was collected, and the solvent was removed by freezing (lyophilization). The dry extract powder was stored in a light-proof container at −20°C and dissolved in distilled water before use.

### 2.2. Polyphenolic Compound Determination of SA Extract

The polyphenolic compounds in SA extract were measured by using reversed-phase high performance liquid chromatography (RP-HPLC) based on the method of Peñarrieta et al. [12]. In brief, a reversed-phase column and a diode array detector (HPLC-DAD) method was followed for the determination of polyphenolic compounds in SA extract using ten standards, including gallic acid, eriodictyol, apigenin, isoquercetin, kaempferol, quercetin, hydroquinone, rutin, catechin, and tannic acid, of phenolic compounds which were chromatographed by HPLC using a UV-vis diode array detector. The detector was set at 280 nm mainly for phenolic acids and was set at 360 nm for flavonoids.

### 2.3. Animals

Thirty adult male zebrafish (*Danio rerio*) with body length 3–5 cm and weight 500 mg were cultured in an acrylic tank with reverse osmosis water and oxygen, keeping the water in the tank for 24 hours before use. The density was set at 5 fish per 7.5 litres. Water temperature and pH were maintained at 28 ± 2°C and 6.5–7.5, respectively, with 12-hour light to dark cycle. The fish were fed with Artemia twice a day (morning-evening). Before the experiment, fish were kept in holding tanks for 7 days. The zebrafish were weighed before feeding. The fish were placed on a damp cloth and then were fed by using a plastic catheter connected with a micropipette. The zebrafish were divided into 6 groups (*n* = 5).

  Group I: control (fed by water)
   Group II: SCO (induced by scopolamine)
   Group III: RV + SCO (induced by scopolamine and fed with rivastigmine 1.5 mg/kg)
   Group IV: SA200 + SCO (induced by scopolamine and fed with SA 200 mg/kg)
   Group V: SA400 + SCO (induced by scopolamine and fed with SA 400 mg/kg)
   Group VI: SA800 + SCO (induced by scopolamine and fed with SA 800 mg/kg)

Each group received the dosages above for 7 days before the behavioural test. All experiments were carried out according to the guidelines of the Institute of Animals for Scientific Purposes Development (IAD). Animal ethics no. UP-AE62-01-04-028 was approved by the Laboratory Animal Research Center, University of Phayao.

### 2.4. Apparatus of Behavioural Tests

The experimental apparatus consisted of a colour-biased appetite conditioning T-maze test and inhibitory avoidance test apparatus following Maddula et al. [13]. The tank for the colour-biased appetite conditioning T-maze test was prepared as an acrylic glass T-shaped maze, dimensions 50 cm × 10 cm × 10 cm for the long arm, and dimensions 20 cm × 10 cm × 10 cm for two short arms. A tank for the inhibitory avoidance test, an acrylic glass with aquarium dimensions of 18 cm × 9 cm × 7 cm, was divided into two equal white and dark compartments by a sliding door. An electrode plate connected with an electrical stimulator was placed in the dark compartment.

### 2.5. Experimental Procedures

**Figure 1:**
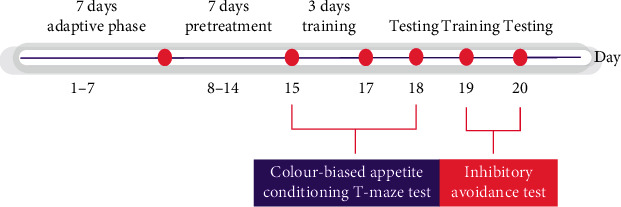
The experimental design of this study.

The experimental procedures followed Maddula et al. [13] with slight adaptions. The zebrafish were fed by rivastigmine and the SA extract 2 hours before the start of the experiment, and 1 hour before the experiment the fish were treated with a 200 *μ*M scopolamine solution [13]. The standard and the extract solutions were freshly prepared on the day of the experiment. The experimental study was done for a total of 20 days; the experimental design is shown in Figure 1.

### 2.6. Colour-Biased Appetite Conditioning T-Maze Test

In the training session, zebrafish were placed in the terminal of long arm. 1 minute after that, the sliding door opened to allow the fish to swim towards the short arms. Once the fish entered any of the short arms, another sliding door at the junction was closed. The fish were authorized to swim in the short arms and were observed for 4 minutes. If the fish swam to the green arm, it was fed by food. If the fish swam to the red arm, it was returned to repeat training for 3 days [13, 14]. During the testing session, fish entered the test by performing the same test as the training process without food in the green arm and with no punishment in the red arm. Time spent and total number of entries into the green arm and red arm were evaluated.

### 2.7. Inhibitory Avoidance Test

In the training day, zebrafish were placed in the white compartment. 1 minute after that, the sliding door opened 1 cm above the tank floor and the fish were authorized to enter the dark compartment. The sliding door was suddenly closed after the zebrafish entered the dark compartment; then, a 3 mA shock current was applied for 2 seconds. In the next day (testing session), zebrafish were tested following the same procedure as the training session without an electrical shock. The latency time to enter the dark compartment was investigated in both the training and testing sessions.

### 2.8. Statistical Analysis

Statistical analysis was performed using SigmaPlot (version 12.0). All values were expressed as mean ± SEM and analysed using one-way ANOVA and Tukey's post hoc tests. Differences between groups were considered statistically significant at *p* value <0.05.

## 3. Results

### 3.1. Polyphenolic Compound Determination of SA Extract

Many polyphenolic compounds were found in SA extract, including flavonoids (isoquercetin, rutin, quercetin, and catechin), phenolic acid (gallic acid), and tannin (tannic acid). The contents of gallic acid, isoquercetin, quercetin, rutin, catechin, and tannic acid in SA extract are shown in Table 1, and the chromatogram of the standard and SA extract is shown in Figures S1 and S2.

### 3.2. Colour-Biased Appetite Conditioning T-Maze Test

**Figure 2:**
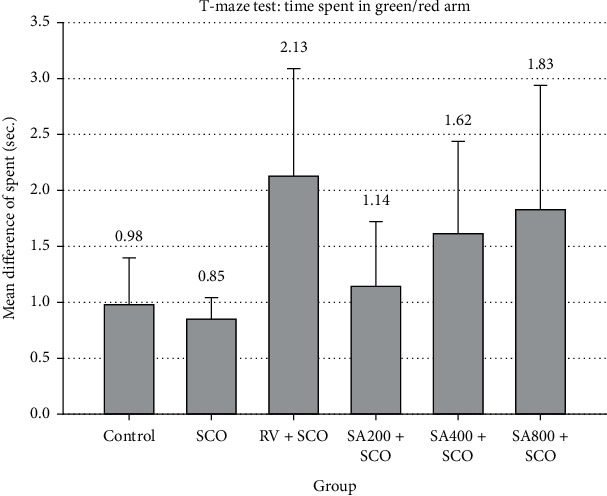
Effect of SCO, RV, and SA extract on time spent in the colour-biased appetite conditioning T-maze test. Mean difference of time spent in green arm and red arm expressed as mean ± SEM. Zebrafish that received RV + SCO, SA200 + SCO, SA400 + SCO, and SA800 + SCO showed mean difference of time spent at more than 1 as 2.13 ± 0.96, 1.14 ± 0.58, 1.62 ± 0.82, and 1.83 ± 1.11, respectively.

**Figure 3:**
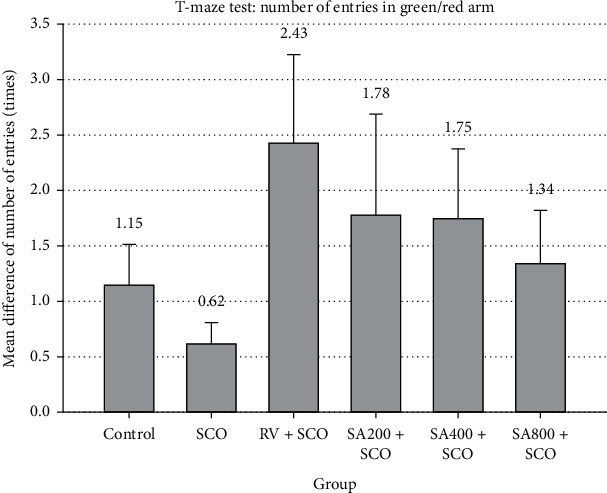
Effect of SCO, RV, and SA extract on number of entries in the colour-biased appetite conditioning T-maze test. Mean difference of total number of entries into green arm and red arm expressed as mean ± SEM. Zebrafish that received RV + SCO, SA200 + SCO, SA400 + SCO, and SA800 + SCO showed mean difference of total number of entries at more than 1 as 2.43 ± 0.80, 1.78 ± 0.91, 1.75 ± 0.63, and 1.34 ± 0.48, respectively.

The time spent and total number of entries in the green arm and red arm test were calculated to find the mean difference (green arm/red arm). For groups with a mean difference of time spent and total number of entries more than 1, it was interpreted that zebrafish spent more time in the green arms than red arms, suggesting that zebrafish in these groups showed good memory performance. Zebrafish that received RV and SA extract at all doses showed mean difference of time spent and total number of entries at more than 1 (Figures 2 and 3, respectively); however, results were not statistically significant.

Zebrafish that received SA extract of 200 mg/kg, 400 mg/kg, and 800 mg/kg recorded longer time spent ratio and greater total number of entries ratio than the control and SCO groups. However, there were no statistically significant differences between the results.

### 3.3. Inhibitory Avoidance Test

**Figure 4:**
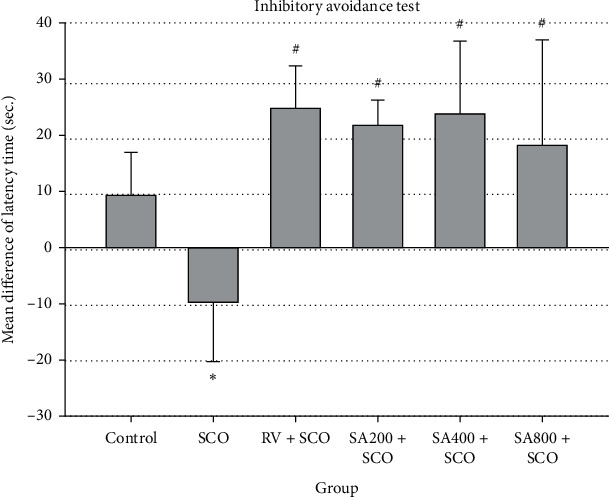
Effect of SCO, RV, and SA extract on the inhibitory avoidance test. Mean difference of latency time in inhibitory avoidance test expressed as mean ± SEM. ^*∗*^*p* value <0.05 compared to the control group, #*p* value <0.05 compared to the SCO group.

In the inhibitory avoidance test, zebrafish that received SA at doses of 200, 400, and 800 mg/kg had significantly increased latency time as 21.75 ± 4.59, 23.75 ± 13.01, and 18.20 ± 18.84 min, respectively, compared to the SCO group (−9.80 ± 10.45 min) (Figure 4). In addition, there were no statistically significant differences among doses of SA. These results suggested that SA at all doses was effective against memory impairment induced by scopolamine, especially fear memory in zebrafish.

## 4. Discussion

The effect of *Streblus asper* extract in preventing dementia induced in zebrafish by scopolamine was investigated. Scopolamine is a high-affinity muscarinic receptor antagonist that binds to acetylcholine on the postsynaptic muscarinic receptor. This increases AChE activity in the cortex and levels of oxidative stress and proinflammatory cytokines in the hippocampus, as well as increased levels of APP and Tau by producing amyloid beta to induce neurodegeneration that can cause Alzheimer's disease. Scopolamine can induce passive avoidance responses and impact the duration of responses to learning in zebrafish. Therefore, we used scopolamine as a toxin to induce zebrafish memory impairment in this study [15]. We also used rivastigmine, an acetylcholinesterase inhibitor, as a positive control to replicate the treatment of Alzheimer's disease [16].

Behavioural testing is widely used to evaluate the effect on learning and memory of zebrafish. Methods include the colour-biased appetite conditioning T-maze test and the inhibitory avoidance test. Spatial memory testing using the colour-biased appetite conditioning T-maze test is related to the lateral pallium located in the telencephalon in zebrafish that is similar to the hippocampus in mammals for remembering the learning process [17]. Memory defects are caused by A*β* accumulation, resulting in the brain's performance gradually decreasing and ACh decreasing. The hippocampus plays an important role in remembering learning information. When these brain cells are destroyed, problems occur with memory, especially short-term memory that affects learning and behaviour. The T-maze test that affects learning and memory of zebrafish was available in 4 colours as blue, green, red, and yellow. In these 4 colours, zebrafish had a higher response to red and green than other colours [18]. Therefore, red and green were used in our learning and recognition experiment for zebrafish, with feeding on the green arm to increase learning and memory called synaesthesia. The T-maze test used time spent in the green arm and red arm as an indicator for testing the learning and memory of zebrafish [18]. This study showed that time spent and total number of entries ratio at more than 1 represented that zebrafish showed good memory performance. The rivastigmine group recorded the highest time spent ratio and total number of entries ratio, suggesting that rivastigmine was more effective in spatial learning and recognition than the other groups due to pharmacological activity that can inhibit AChE activity.

Fear memory using the inhibitory avoidance test involved the medial pallium of zebrafish. This has a similar function to the amygdala in the human brain. The inhibitory avoidance test measured the latency time to enter a dark compartment to evaluate the effect of SA extract on the behaviour of learning and memory. Results were expressed as the mean difference of latency time between testing and training days, showing that rivastigmine and SA extract at all doses significantly increased the mean difference of latency time compared with the SCO group. However, there were no statistically significant differences between the SA extract and rivastigmine groups.

Preclinical animal models are necessary tools for behavioural testing research. It is important to test hypotheses and to assess the potential of new therapeutic agents. However, there are reports of the limitations of behaviour testing research which are related to the following factors: the experiment environment, location, noise, vibration, and the behavioural task itself [19]. Therefore, this is the possible cause that no significant difference was seen in the T-maze test, and increasing the sample size of fish used could yield more insight.

It is widely accepted that SA possesses analgesic, anti-inflammatory, antioxidant, and anti-Parkinson activities, with possible clinical applications in many disorders [8, 9, 20]. SA extract contains important phytochemicals as flavonoids such as quercetin, isoquercetin, and rutin as antioxidant compounds. Li and Pu and Zhang et al. reported that the neuroprotective effects of flavonoids might be due to a reduction in oxidative stress [21, 22]. SA extract also inhibited AChE enzyme activity and prevented the occurrence of neuroinflammatory and oxidative stress [10]. Our findings demonstrated that SA leaf extracts show potential for the prevention and treatment of memory impairment or Alzheimer's disease.

## 5. Conclusions

The effects of SA leaf extract on scopolamine-induced memory deficit in zebrafish were investigated. Results showed that SA leaf extract attenuated memory impairment in an inhibitory avoidance test related to fear memory. Our findings can be useful for further research to develop SA extract as a health product to ameliorate memory impairment or Alzheimer's disease in the future.

## Figures and Tables

**Table 1:** The contents of polyphenolic compounds in SA extract.

Polyphenolic compound	Contents (mg/kg extract)
Gallic acid	837.71
Isoquercetin	202.47
Quercetin	137.57
Rutin	443.57
Catechin	204.59
Tannic acid	1,137

## Data Availability

The data that support the findings of this study are available from the corresponding author upon reasonable request.

## Supplementary Materials

Supplementary Materials Figure S1 Peak of HPLC chromatogram of the standards, including gallic acid, eriodictyol, apigenin, isoquercetin, kaempferol, quercetin, hydroquinone, rutin, catechin, and tannic acid. Figure S2 Peak of HPLC chromatogram of the SA extract.
