# Supplementary material for: Effect of Streblus asper Leaf Extract on Scopolamine-Induced Memory Deficits in Zebrafish: The Model of Alzheimer's Disease
Source: Adv Pharmacol Pharm Sci. 2021 Apr 24;2021:6666726. doi: 10.1155/2021/6666726 (PMC8093034; doi:10.1155/2021/6666726)
Supplement: Supplementary Materials — Figure S1 Peak of HPLC chromatogram of the standards, including gallic acid, eriodictyol, apigenin, isoquercetin, kaempferol, quercetin, hydroquinone, rutin, catechin, and tannic acid. Figure S2 Peak of HPLC chromatogram of the SA extract. [file 6666726.f1.docx]

# Effect of *Streblus asper* leaf extract on scopolamine-induced memory deficits in a zebrafish: The model of Alzheimer’s disease.

# Running Title

# Anti-memory deficits of *Streblus asper* leaf extract

Kanathip Singsai^1,2^, Natthanicha Ladpala^1^, Natthan Dangja^1^, Thanyaret Boonchuen^1^, Niracha Jaikhamfu^1^, Pirinyapat Fakthong^1^

^1^ Department of Pharmaceutical care, School of Pharmaceutical Sciences, University of Phayao, Phayao, Thailand, 56000

^2^ Unit of Excellence of Pharmacological Research and Vaccine Development in Animal Models, University of Phayao, Phayao, Thailand, 56000

Correspondence should be addressed to Kanathip Singsai; [kanathip.si@up.ac.th](mailto:kanathip.si@up.ac.th)

**-Supplementary data-**

**Polyphenolic compound determination of SA extract**

Many polyphenolic compounds were found in SA extract, including flavonoids (isoquercetin, rutin, quercetin, and catechin), phenolic acid (gallic acid) and tannin (tannic acid). The chromatogram of the standard and SA extract showed as Figure 1 and 2, respectively.

**
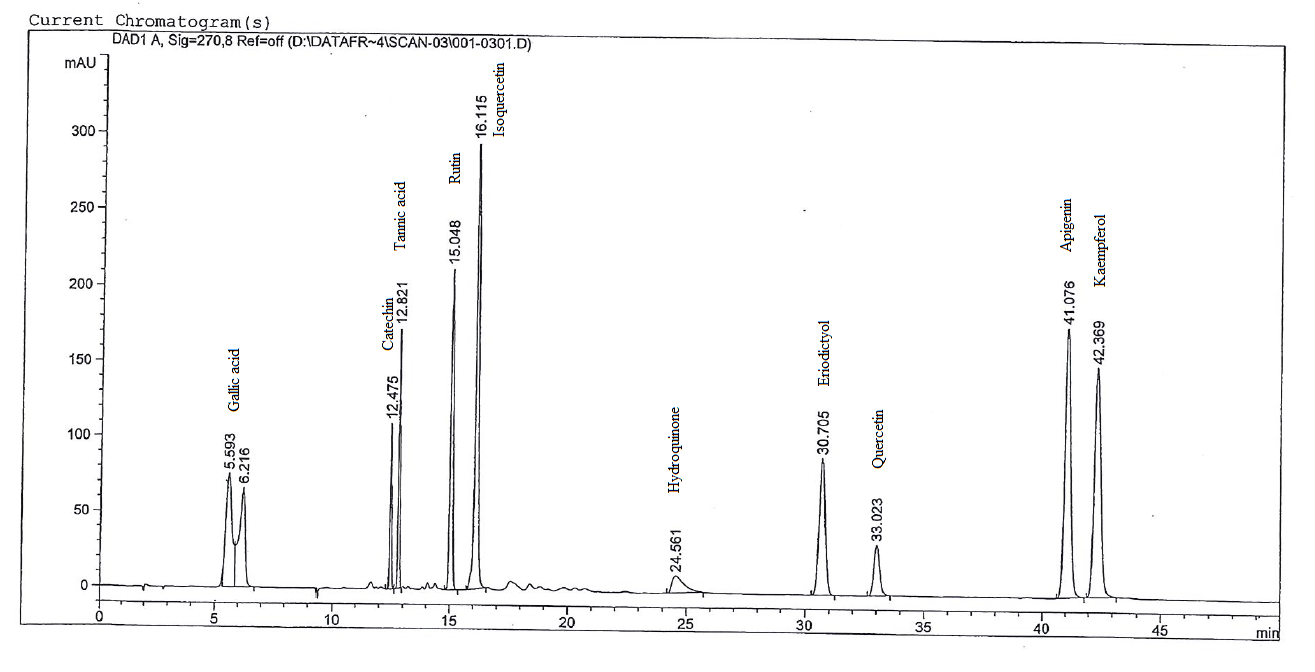
**

**Figure 1.** Peak of HPLC chromatogram of the standards, including gallic acid, eriodictyol, apigenin, isoquercetin, kaempferol, quercetin, hydroquinone, rutin, catechin, and tannic acid.

**
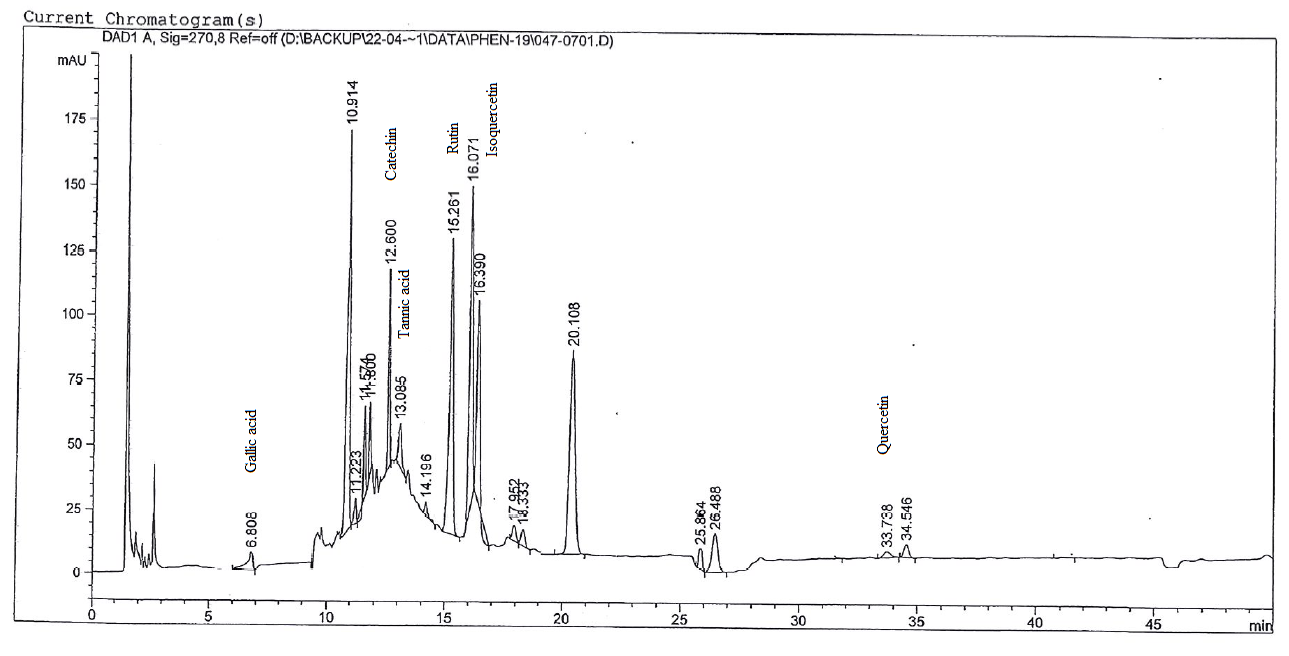
**

**Figure 2.** Peak of HPLC chromatogram of the SA extract.
